# Transthoracic coronary flow reserve and dobutamine derived myocardial function: a 6-month evaluation after successful coronary angioplasty

**DOI:** 10.1186/1476-7120-2-26

**Published:** 2004-12-06

**Authors:** Silvana Cicala, Maurizio Galderisi, Pasquale Guarini, Arcangelo D'Errico, Pasquale Innelli, Moira Pardo, Giancarlo Scognamiglio, Oreste de Divitiis

**Affiliations:** 1 Cardioangiology Unit, Department of Clinical and Experimental Medicine, Federico II University Hospital Naples, Italy; 2 Division of Cardiology, "Villa dei Fiori" Hospital Naples, Italy

**Keywords:** Percutaneous coronary angioplasty, Coronary flow reserve, Color Tissue Doppler, Stress-echo

## Abstract

After percutaneous transluminal coronary angioplasty (PTCA), stress-echocardiography and gated single photon emission computerized tomography (g-SPECT) are usually performed but both tools have technical limitations. The present study evaluated results of PTCA of left anterior descending artery (LAD) six months after PTCA, by combining transthoracic Doppler coronary flow reserve (CFR) and color Tissue Doppler (C-TD) dobutamine stress.

Six months after PTCA of LAD, 24 men, free of angiographic evidence of restenosis, underwent standard Doppler-echocardiography, transthoracic CFR of distal LAD (hyperemic to basal diastolic coronary flow ratio) and C-TD at rest and during dobutamine stress to quantify myocardial systolic (S_m_) and diastolic (E_m _and A_m_, E_m_/A_m _ratio) peak velocities in middle posterior septum. Patients with myocardial infarction, coronary stenosis of non-LAD territory and heart failure were excluded. According to dipyridamole g-SPECT, 13 patients had normal perfusion and 11 with perfusion defects. The 2 groups were comparable for age, wall motion score index (WMSI) and C-TD at rest. However, patients with perfusion defects had lower CFR (2.11 ± 0.4 versus 2.87 ± 0.6, p < 0.002) and septal S_m _at high-dose dobutamine (p < 0.01), with higher WMSI (p < 0.05) and stress-echo positivity of LAD territory in 5/11 patients. In the overall population, CFR was related negatively to high-dobutamine WMSI (r = -0.50, p < 0.01) and positively to high-dobutamine S_m _of middle septum (r = 0.55, p < 0.005).

In conclusion, even in absence of epicardial coronary restenosis, stress perfusion imaging reflects a physiologic impairment in coronary microcirculation function whose magnitude is associated with the degree of regional functional impairment detectable by C-TD.

## Introduction

Percutaneous transluminal coronary angioplasty (PTCA) has deeply modified the effective management of coronary artery disease [1]. Coronary artery restenosis is unfrequent when PTCA is associated to coronary stenting application which is able to enlarge the lumen area stenosis [2,3]. However, even in absence of coronary artery restenosis, the results of revascularization can be suboptimal because of coronary microvessel dysfunction subsequent to the procedure [4,5]. This issue may be intriguing for management of patients undergone PTCA.

The non-invasive assessment after PTCA is usually performed by gated single photon emission computerized tomography (g-SPECT) [6,7] and by stress echocardiography [7,8]. However, both these tools present technical limitations, g-SPECT having a low specificity [9] and semi-quantitative echocardiographic wall motion analysis low sensitivity [10].

In the last years, great interest has been developed about new echocardiographic techniques as Doppler-derived coronary flow reserve (CFR) [11,12] and color Tissue Doppler (C-TD) [13,14]. The first tool provides reliable information about coronary microvascular function in absence of epicardial coronary stenosis [15] while C-TD is able to quantify left ventricular (LV) myocardial performance both at rest and during pharmacological stress [13,14].

On these grounds, aim of the present study was to assess C-TD derived myocardial performance, both at rest and during pharmacologic stress, in relation to the function of coronary microcirculation determined by non invasive CFR after successful PTCA of LAD.

## Methods

### Study population

Among 30 patients who had undergone PTCA with stenting for significant LAD stenosis between September and October 2000, 24 patients (age = 50–64 years) free of coronary angiographic evidence of LAD restenosis 6 months after the procedure, entered the study and performed non-invasive test screening in the same period of the invasive assessment (±7 days). The informed consent of all patients and approval of Institutional Committee were obtained. Patients were excluded for acute and previous myocardial infarction (according to ECG at rest), concomitant coronary stenosis of right coronary artery and/or circumflex artery, congestive heart failure, valvular heart disease, primary cardiomyopathy, atrial fibrillation, inadequate quality echocardiograms. On the basis of g-SPECT dipyridamole induced perfusion defects, the study population was divided into 2 groups: without and with perfusion defects.

### Procedures

Patients underwent dipyridamole gated myocardial perfusion g-SPECT acquisition, transthoracic echocardiography, C-TD (both at baseline and during dobutamine stress) and non-invasive CFR determination by dipyridamole test. All echocardiographic measurements were analyzed without knowledge of the clinical data. According to the rules of the Institutional Committees, all patients withdrew cardiac drugs at least 2 days before the performance of the non invasive assessment.

### Dipyridamole g-SPECT

Single day rest/dipyridamole g-SPECT was performed according to the standard methods by injecting patients with technetium 99m (^99m^Tc) tetrophosmin 8 mCi (296 MBq) at rest and 24 mCi (888 MBq) after dipyridamole infusion by volumetric pump (dose of 0.14 mg/kg/min in 4 minutes) [16,17]. A single stress SPECT corresponds to a dose exposure of about 500 chest x-ray. Qualitative assessment of reconstructed gated images was obtained on mid-short axis slices, vertical and horizontal long axis slices.

#### Transthoracic Echocardiography

Standard echocardiographic examinations were performed using a System FiVe, Vingmed Sound AB machine (GE, Horten, Norway), by a 2.5 MHz transducer equipped with second harmonic capability. M-mode echocardiographic analysis was performed according to the criteria of the American Society of Echocardiography [18] and LV mass indexed for height powered to 2.5 [19]. LV end-diastolic and end-systolic volumes were estimated according to the Simpson method [20] and LV ejection fraction derived.

#### Stress protocols

Dobutamine stress protocol was performed according to the standard method [21] using low and high-dose (up to 40 μg/Kg/min) by using the System FiVe Vingmed machine. C-TD of posterior septum was recorded at rest and during each dobutamine stage. CFR assessment was performed by HDI 5000 ultrasound machine (ATL Ultrasound, Bothell, Washington, USA), using a high-frequency (7 MHz) transducer. The visualization of the distal portion of the left anterior descending artery and the recording of PW-Doppler derived coronary blood flow velocities performed at baseline and after dipyridamole infusion (0.56 mg/kg over 4 minutes) according to the standards of our laboratory [14]. Blood pressure and a 12-lead ECG were recorded at rest and at the end of each stage of both dobutamine and dipyridamole tests.

#### C-TD dobutamine stress echocardiography analysis

Echocardiographic images were recorded on S-VHS videotapes and digitally stored on magneto-optical disk for subsequent analysis. Images were evaluated by 2 experienced observers. Baseline and stress wall motion analysis was performed by 2 experienced readers blinded to the other data. Regional wall motion was assessed with a 16-segment model of the left ventricle and semiquantitatively graded from 1 to 4 as follows: 1 = normal; 2 = hypokinesia; 3 = akinesia; and 4 = dyskinesia. A wall motion score index (WMSI), obtained dividing the sum of each segment scores by the number of the segments, was assessed both at baseline and at high-dose dobutamine.

C-TD acquisition of posterior septal wall was performed in real time, superimposed on 2-D images, at baseline and at the end of each dobutamine infusion stage. C-TD imaging was stored in digital format and analyzed off line on cine-loop as previously described [14]. The region of interest was the middle segment of the posterior septum where myocardial velocity profile was obtained. The middle posterior septum for measurements of C-TD was chosen since the perfusion of this myocardial segment is provided by a branch of LAD, where also CFR was determined. The reproducibility of C-TD of our laboratory has been reported, the intra- and inter-observer variability being <3% and <6% for all the measurements both at rest and at high-dose dobutamine [22].

#### CFR Analysis

Methods and reproducibility (intra-observer and inter-observer variability 1.9% and 4.2% respectively) of our laboratory in measuring coronary blood flow reserve has been described [22]. By placing sample volume on the color signal, spectral Doppler of LAD flow showed the characteristic biphasic flow pattern with a larger diastolic and a smaller systolic component. Diastolic peak velocities were measured at baseline and after dipyridamole, by averaging the highest 3 spectral Doppler signals for each measurement. CFR was defined as the ratio of hyperemic to basal diastolic peak velocities. All images were recorded on a magneto-optical disk and analyzed off-line by 2 independent observers, blinded to the other data.

### Statistical Analysis

The analyses were performed by SPSS for Windows release 8.0 (Chicago, Illinois, USA). Data are presented as mean value ± SD. Analysis of variance was used to assess intergroup differences. Linear regression analyses and partial correlation test was done using Pearson's method. Differences were considered significant at p < 0.05.

## Results

### Characteristics of the study population

The characteristics of the study population and both heart rate and blood pressure at baseline and at high-dose dobutamine are listed in Table 1. The 2 groups were comparable for heart rate and blood pressure values both at rest and at stress dobutamine peak. Of note, the prevalence of arterial hypertension, diabetes mellitus, hypercholesterolemia and smoke was not different between groups and no patients of both groups presented g-SPECT derived myocardial perfusion defects at rest (data not reported in Table).

**Table 1:** Characteristics of the study population

**Variable**	**Normal Perfusion **n = 13	**Perfusion Defect **n = 11	**P**
Age (years)	55.9 ± 4.1	58.4 ± 3.1	NS
Body mass index (Kg/m^2^)	26.1 ± 1.1	26.3 ± 0.8	NS
Baseline Systolic BP (mm Hg)	147.0 ± 7.5	149.2 ± 11.6	NS
Baseline Diastolic BP (mm Hg)	85.1 ± 7.5	85.5 ± 9.1	NS
Baseline Heart rate (bpm)	74.8 ± 5.9	73.7 ± 6.9	NS
DOB Systolic BP (mm Hg)	151.1 ± 7.5	151.9 ± 10.2	NS
DOB Diastolic BP (mm Hg)	83.2 ± 6.3	83.4 ± 7.3	NS
DOB Heart rate (bpm)	139.6 ± 5.4	141.0 ± 5.5	NS

BP = Blood Pressure, DOB = Dobutamine

### Echocardiographic analysis

The comparisons of echocardiographic measurements and CFR between the 2 groups are reported in Table 2. Because of higher septal and posterior wall thickness, patients with myocardial perfusion defects after PTCA had greater LV mass index (p < 0.05). LV ejection fraction was comparable between the 2 groups.

**Table 2:** Standard Doppler echocardiographic and CFR analysis

**Variable**	**Normal Perfusion**	**Perfusion Defect**	**P**
Septal wall thickness (mm)	10.1 ± 1.4	11.2 ± 0.4	<0.02
Posterior wall thickness (mm)	10.2 ± 1.4	10.6 ± 0.5	NS
LV internal diastolic diameter (mm)	54.7 ± 2.6	56.7 ± 3.5	NS
LV internal systolic diameter (mm)	39.2 ± 2.8	39.6 ± 2.9	NS
2-D Ejection Fraction (%)	54.8 ± 6.0	54.9 ± 3.4	NS
LV mass index (g/m ^2.7^)	49.6 ± 10.7	57.9 ± 8.0	<0.05

LV = left ventricular

### Dobutamine test and Color TD analysis

WMSI was comparable between the 2 groups at baseline (1.07 ± 0.10 versus 1.15 ± 0.11) whereas it was higher at low-dose dobutamine (1.07 ± 0.11 versus 1.17 ± 0.12) and at high-dose dobutamine (1.07 ± 0.12 versus 1.20 ± 0.14) (both p < 0.05) in patients with SPECT-derived perfusion defects than in controls. Positive dobutamine stress-echo involving LAD territory (and in particular mid-septal region) was observed in 5/11 patients (45.4%) with SPECT perfusion defects.

**Figure 1:**
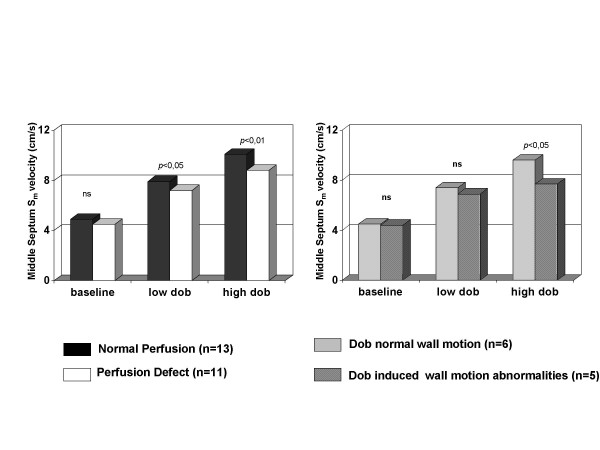
In the left panel comparison of S_m _peak velocity of middle posterior septum of patients without and with SPECT perfusion defects at rest, at low and at high-dose dobutamine. In the right panel comparison of S_m _peak velocity of middle posterior septum during dobutamine stress echocardiography in patients with perfusion defects having or not dobutamine-induced wall motion abnormalities.

C-TD diastolic measurements of mid-septum (E_m_, A_m_, E_m_/A_m _ratio) were similar between the two groups at rest (E_m_/A_m _ratio = 1.04 ± 0.1 and 1.03 ± 0.3 in patients with and without perfusion defects respectively, NS) and at low dose dobutamine (E_m_/A_m _ratio = 1.00 ± 0.1 and 1.13 ± 0.4 respectively, NS) while E_m_/A_m _ratio was mildly different at high-dose dobutamine (0.83 ± 0.2 and 0.70 ± 0.2 respectively, p < 0.05). S_m _peak velocities were lower in patients with perfusion defects at low- (p < 0.05) and at high-dose dobutamine (p < 0.01) and were significantly lower also in patients with perfusion defects showing stress induced wall motion abnormalities in comparison with patients with perfusion defects but no change of wall motion during dobutamine infusion (Figure 1).

### CFR analysis

Coronary diastolic peak velocities were similar at rest between the two groups (21.5 ± 5.2 cm/s in patients without perfusion defects and 22.0 ± 19 cm/s in patients with perfusion defects, NS) but significantly different after dipyridamole (60.0 ± 16.1 cm/s versus 49.1 ± 10.8 cm/s respectively, p < 0.05). Thus, CFR was 2.87 ± 0.6 in patients without defects and 2.11 ± 0.4 in patients with perfusion defects (p < 0.002). Of note, analyzing the group with SPECT-derived perfusion defects, the patients with stress inducible wall motion abnormalities had lower CFR (1.91 ± 0.1) than those without change of WMSI during dobutamine infusion (2.28 ± 0.4) (p = 0.06).

#### Relationship between CFR and Dobutamine stress measurements

**Figure 2:**
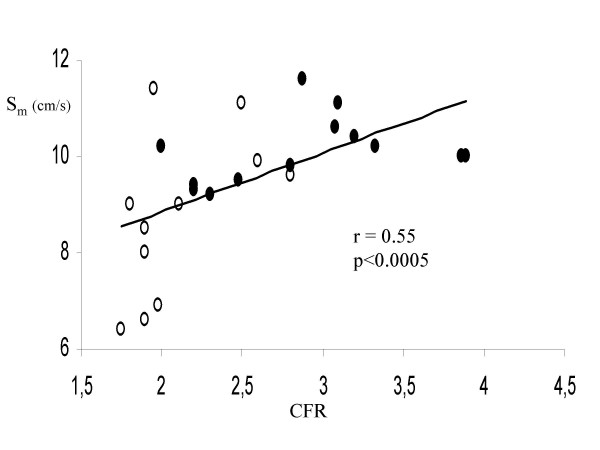
Positive association between CFR and C-TD derived S_m _peak velocity of middle septum at high-dose dobutamine. Full circles indicate patients with SPECT-derived myocardial perfusion defects; empty circles indicates patients without perfusion defects.

**Figure 3:**
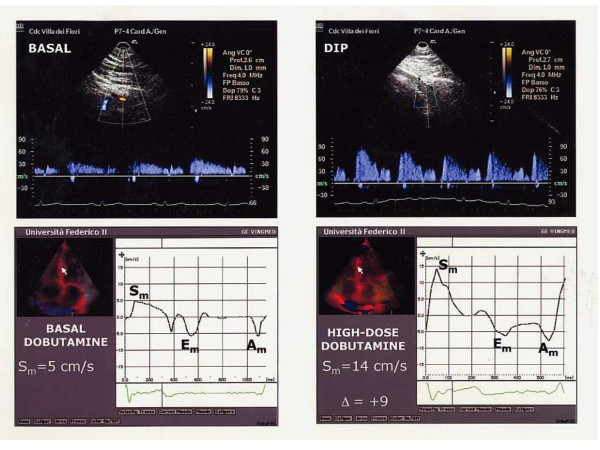
CFR and S_m _peak velocity of middle septum at high-dose dobutamine in a patient with SPECT derived normal perfusion. The upper panels show coronary artery flow velocity in the LAD at baseline and with a normal increase with dipyridamole (DIP). In the lower panels, myocardial systolic velocity (S_m_) shows a normal increase at high-dose dobutamine.

**Figure 4:**
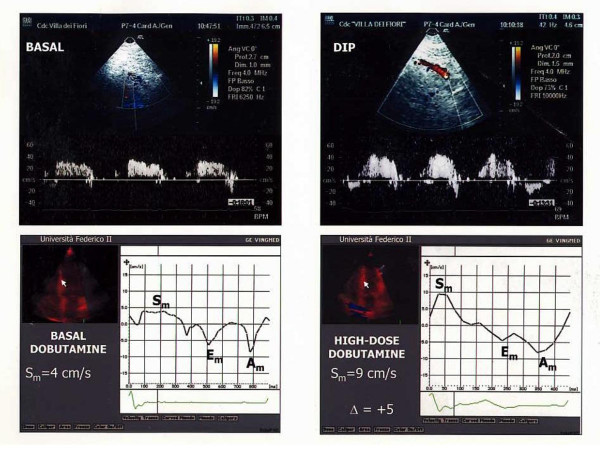
CFR and septal S_m _peak velocity at high-dose dobutamine in a patient with SPECT derived perfusion defect. The upper panels display a reduced CFR. In the lower panels, the increase of septal S_m _from baseline to high-dose dobutamine is low.

In the overall population, CFR was negatively related to WMSI at low-dose (r = -0.46, p < 0.02) and high-dose dobutamine (r = -0.50, p < 0.01). Among C-TD Doppler measurements, CFR was positively related to S_m _peak velocity of middle septum at low dose (r = 0.39, p < 0.05) and high-dose dobutamine (r = 0.55, p < 0.0005) (Figure 2) while the relation of S_m _at baseline (r = 0.12) did not achieve the statistical significance. No relation of CFR was found with C-TD diastolic measurements of middle septum at any stage of dobutamine stress. Figure 3 shows a patient with SPECT derived normal perfusion: CFR is >2 and middle septal S_m _peak velocity has a significant increment from baseline to high-dose dobutamine (Δ = +9). Figure 4 displays a patient with a perfusion defect: CFR is reduced and middle septal S_m _increase from baseline to high-dose dobutamine is lower (Δ = +5).

## Discussion

The present study used new ultrasound tools, as off-line quantitative C-TD [13,14] and Doppler-derived CFR [15], to evaluate long-term effects of PTCA on the relation between myocardial performance and coronary microvascular function, in the absence of angiographic coronary artery restenosis. According to dipyridamole g-SPECT, the population was divided into 2 groups, the first without and the second one with perfusion defect in the LAD territory, comparable for resting LV ejection fraction. Our findings show that, six months after successful PTCA of LAD, patients with myocardial perfusion defects present both lower CFR and reduced peak dobutamine myocardial systolic function of wall involved by LAD perfusion (i.e., middle posterior septum) in comparison with the control group and that CFR is positively related to stress peak S_m _velocity measured at middle septum in the overall population.

### CFR and perfusion defect after PTCA

According to the study design, we intentionally selected patients without angiographic evidence of post-PTCA LAD restenosis. Worthy of note, the incidence of coronary restenosis was very low 6 months after the procedure, in accord with previous experiences about the combined use of stenting and PTCA [23]. Nevertheless, 11 our patients without restenosis showed dipyridamole g-SPECT LAD perfusion defects. Myocardial hypoperfusion may occur in patients without overt epicardial coronary artery stenosis having coronary microvessel damage [24,25] and coronary microvascular dysfunction corresponds to a reduced CFR in the absence of epicardial coronary stenosis [26]. Accordingly, the patients of the present study with long-standing SPECT perfusion defects showed lower CFR than the control group. An abnormal CFR had been already described immediately after balloon angioplasty [5,27,28], probably because of a slow recovery of autoregulation in the microvascular bed [29]. This reduction is primarily due to an increased flow velocity at rest [5,27,28], in relation to the failure of microvessel bed to vasoconstrict appropriately and/or to epicardial vasoconstriction mediated by a myogenic response and/or neural mechanism [30]. In contrast to previous studies showing normalization of CFR after three [31], five [32] or six months [5], CFR was persistently reduced in our patients with SPECT-derived perfusion defects. A suboptimal Doppler flow wire derived CFR had been observed six months after PTCA without restenosis by DEBATE investigators [33]. In the suboptimal CFR group the reduction of CFR was mainly due to a long-standing elevation in resting peak velocities while in our patients with perfusion defects it was due to a blunted maximal vasodilator response to dypiridamole. It is conceivable that this alteration could depend on endothelial damage of coronary microcirculation [34] preceding the procedure and persisting long time after PTCA. Coronary microcirculatory vasoconstriction induced by endothelial dysfunction has been described as effect of spontaneous myocardial ischemia [35] as well as in conditions other than epicardial coronary artery stenosis, as diabetes mellitus [36,37] arterial hypertension [26,38] and LV hypertrophy [39,40], which can alter microvascular function. However, an alternative interpretation of our findings include the possibility that a residual coronary stenosis might be anatomically insignificant but hemodynamically important, thus explaining a discrepancy between the percentage of lumen reduction and the amount of regional flow reserve.

### Myocardial systolic function and perfusion defect after PTCA

The reduction of myocardial systolic function expressed by the decrease of low and high-dose dobutamine S_m _in middle septum, i.e. in the territory supplied by LAD, is not surprising in patients with perfusion defects after PTCA. Of note, myocardial systolic performance of middle septum was not significantly different between the 2 groups at rest. These findings are consistent with an altered myocardial systolic velocity response to exercise already described by C-DT in patients with coronary artery disease [13,41]. Also WMSI, not different at rest, became significantly higher at low and high-dose dobutamine in patients with perfusion defects. This increase (involving LV segments of LAD territory) during stress occurred only in 5/11 patients who had lower S_m _peak velocities at peak dobutamine stress and lower CFR than patients without inducible wall motion abnormalities. Myocardial reperfusion injury may include LV regional systolic dysfunction as irreversible manifestation, it depending by a reduction of myocardial blood flow reserve [5]. Inducible wall motion abnormalities in the presence of a successful coronary revascularization might indicate a very severe microvascular damage.

### Association between CFR and myocardial systolic function

It is recognized that the extent of stress dobutamine-induced dissinergy is associated to the degree of CFR reduction in patients with significant coronary artery stenosis, an invasive myocardial fractional flow reserve ≤0.75 having a sensitivity of 76% and a specificity of 97% [42]. In agreement with these findings, we found a positive association between the functional degree of vasodilator microvascular coronary circulation and the magnitude of regional myocardial systolic function at peak dobutamine stress, i.e. S_m _peak velocity of the wall (middle septum) supplied by LAD. Accordingly, we also found a lower but significant negative relation between CFR and stress peak WMSI. Since patients undergoing successful reperfusion procedures generally present a good stress-echo LV functional response [6], our data suggest the ability of C-DT to detect even minor forms of LV regional myocardial dysfunction occurring under these circumstances.

### Study Limitations

The main limitation of the present study include the fact that the negativity of coronary angiography can not exclude definitely the presence of coronary restenosis while an invasive measurement of CFR by Doppler flow wire or an intra-coronary ultrasound could have been crucial to clarify this issue. Unfortunately, these evaluations were not included into our study protocol. In addition, it has also to be underscored that patients with perfusion defects of the present study had greater LV mass, a factor which can itself induce a reduction of CFR [39,40].

### Clinical implications

The results of the present study suggest that CFR impairment may be detectable after PTCA even in the absence of coronary restenosis, it depending by an altered coronary microvascular function. In this clinical scenario, SPECT stress perfusion defects have to be interpreted as false positive results for PCTA restenosis while they reflect a true physiologic impairment in regional CFR with some associated degree of systolic impairment detectable by C-TD. Quantitative parameters of CFR and C-TD can provide an additive value over conventional stress echocardiographic assessment.

## Competing interests

### Financial competing interests

• In the past five years we didn't receive reimbursements, fees, funding, or salary from an organization that may in any way gain or lose financially from the publication of this manuscript, either now or in the future.

• No organization financied this manuscript.

• We didn't hold any stocks or shares in an organization that may in any way gain or lose financially from the publication of this manuscript, either now or in the future.

• We didn't hold or are currently applying for any patents relating to the content of the manuscript We didn't receive reimbursements, fees, funding, or salary from an organization that holds or has applied for patents relating to the content of the manuscript.

• We don't have any other financial competing interests.

### Non-financial competing interests

There are not any non-financial competing interests (political, personal, religious, academic, intellectual, commercial or any other) to declare in relation to this manuscript.

We have not a competing interest, please discuss it with the editorial office.
